# Neurological Examination via Telemedicine: An Updated Review Focusing on Movement Disorders

**DOI:** 10.3390/medicina60060958

**Published:** 2024-06-09

**Authors:** Efthalia Angelopoulou, Christos Koros, Evangelia Stanitsa, Ioannis Stamelos, Dionysia Kontaxopoulou, Stella Fragkiadaki, John D. Papatriantafyllou, Evangelia Smaragdaki, Kalliopi Vourou, Dimosthenis Pavlou, Panagiotis D. Bamidis, Leonidas Stefanis, Sokratis G. Papageorgiou

**Affiliations:** 1 1st Department of Neurology, Aiginition University Hospital, Vasilissis Sofias Street 72-74, 11528 Athens, Greece; angelthal@med.uoa.gr (E.A.); eva.st.92@gmail.com (E.S.); j-stam@hotmail.gr (I.S.); d.kontaxopoulou@hotmail.com (D.K.); st.fragkiadaki@gmail.com (S.F.); jpapatriantafyllou@gmail.com (J.D.P.); eua_smaragdaki@hotmail.com (E.S.); kalliopivourou@gmail.com (K.V.); lstefanis@bioacademy.gr (L.S.); sokpapa@med.uoa.gr (S.G.P.); 2 School of Topography and Geoinformatics, University of West Attica, Ag. Spyridonos Str., 12243 Aigalew, Greece; dpavlou@central.ntua.gr; 3 Lab of Medical Physics and Digital Innovation, School of Medicine, Aristotle University of Thessaloniki, 54124 Thessaloniki, Greece; bamidis@med.auth.gr

**Keywords:** telemedicine, teleneurology, movement disorders, Parkinson’s disease, neurological examination, tremor; UPDRS, remote examination, video, COVID-19

## Abstract

Patients with movement disorders such as Parkinson’s disease (PD) living in remote and underserved areas often have limited access to specialized healthcare, while the feasibility and reliability of the video-based examination remains unclear. The aim of this narrative review is to examine which parts of remote neurological assessment are feasible and reliable in movement disorders. Clinical studies have demonstrated that most parts of the video-based neurological examination are feasible, even in the absence of a third party, including stance and gait—if an assistive device is not required—bradykinesia, tremor, dystonia, some ocular mobility parts, coordination, and gross muscle power and sensation assessment. Technical issues (video quality, internet connection, camera placement) might affect bradykinesia and tremor evaluation, especially in mild cases, possibly due to their rhythmic nature. Rigidity, postural instability and deep tendon reflexes cannot be remotely performed unless a trained healthcare professional is present. A modified version of incomplete Unified Parkinson’s Disease Rating Scale (UPDRS)-III and a related equation lacking rigidity and pull testing items can reliably predict total UPDRS-III. UPDRS-II, -IV, Timed “Up and Go”, and non-motor and quality of life scales can be administered remotely, while the remote Movement Disorder Society (MDS)-UPDRS-III requires further investigation. In conclusion, most parts of neurological examination can be performed virtually in PD, except for rigidity and postural instability, while technical issues might affect the assessment of mild bradykinesia and tremor. The combined use of wearable devices may at least partially compensate for these challenges in the future.

## 1. Introduction

Patients with movement disorders, such as Parkinson’s disease (PD), essential tremor (ET), dystonia syndromes, and rarer diseases like Huntington’s disease (HD) and atypical parkinsonian syndromes, require regular medical evaluation and multidisciplinary care of their symptoms and needs. Atypical cases often necessitate the assessment by movement disorder specialists for an accurate diagnosis, while the objective neurological examination is crucial not only for diagnosis but also the evaluation of response to treatment changes [1]. The symptoms of PD patients with levodopa-induced motor complications may fluctuate during the day and day to day, and their evaluation during the infrequent in-person visits may not be representative of disease severity [2]. Interventional therapies, such as deep brain stimulation (DBS) and levodopa–carbidopa intestinal gel (LCIG) infusion for PD or botulinum toxin injection treatment for dystonia may require the regular follow-up by specialized neurologists in metropolitan areas [2]. PD patients with access to neurologists display higher survival rates, as well as less likelihood of hip fractures and placement in nursing homes [3]. Furthermore, seeing a PD specialist compared to a general neurologist is associated with higher satisfaction with medical care [4]. 

However, individuals living in remote, rural, and underserved regions may face significant challenges in reaching specialized care, due to the uneven distribution of neurologists and movement disorder specialists in particular [5,6]. Financial reasons and extensive waiting lists for an appointment with a neurologist may further limit access to appropriate care [5,6,7]. In the United States, it has been estimated that more than one-third of patients with PD may not have been evaluated by a neurologist [3]. In less wealthy countries, this proportion is probably even higher [8]. As a result, correct diagnosis might be delayed, while drug-induced side effects including dyskinesia, sedation, or psychiatric symptoms might remain untreated for a prolonged time [6]. On the other hand, timely assessment could have reversed many of them through cessation or dose reduction of medication, thereby contributing to a better quality of life [6]. As the disease progresses, patients may be restricted to a wheelchair or become bedridden, which further limits their accessibility to appropriate neurological services [6]. The COVID-19 pandemic created additional challenges for in-person medical visits due to infection risk [9]. 

Telemedicine, defined as the remote delivery of care via information and communications technology, is a rapidly growing field that might significantly improve accessibility to specialized healthcare [10]. The main forms of telemedicine include synchronous (“real-time”, such as interactive videoconferencing) and asynchronous (“store-and-forward”) applications. The neurological assessment of patients with movement disorders is primarily visual, thus making telemedicine an attractive means for the phenomenologic approach, diagnosis, treatment, and follow-up, as well as education and research [8]. Video-captured hyperkinetic or hypokinetic movement disorders can be sent from physicians in underserved areas to academic centers for neurological consultation, and real-time videoconferencing allows for the synchronous interactive examination [11]. Remote web-based follow-up of patients with PD has been associated with high satisfaction rates and reduced transportation and travel costs [12,13]. Approximately half of local physicians would recommend telemedicine visits through videoconferencing for a more effective communication with remote PD specialists [14]. Although the primary focus of studies is placed on PD, accumulating evidence suggests its promising potential in other movement disorders as well. In HD, virtual visits at patients’ homes are feasible [15], and telemedicine can improve access to predictive testing while maintaining quality of care, counselling, and support [16]. Telemedicine visits can also be conducted for patients with cervical dystonia with high satisfaction levels [17]. 

Despite the significant opportunities of remote assessment, the neurological examination is not possible for all of its elements in virtual settings, and it cannot completely replace the face-to-face evaluation [18]. For instance, the assessment of tone, reflexes, and sensation, as well as the precise examination of muscle power, cannot be performed remotely without the assistance of a healthcare professional. The video-based two-dimensional visualization of the three-dimensional clinical features of movement disorders may affect the accuracy of the remote examination due to incomplete information gathering [19]. Of concern, according to a global survey among movement disorder specialists, the most common challenge in the use of telemedicine was the limited neurological examination [20]. The lack of training and possible discomfort of neurologists towards remote neurological examination might constitute a barrier for its use and effective implementation in routine clinical practice [18]. A qualitative study indicated that during the COVID-19 pandemic, neurologists exhibited low levels of confidence regarding their clinical decisions via telemedicine, and additional in-person visits were sometimes required [21]. During the last three years, a growing number of studies have investigated the feasibility and validity of remote neurological assessment, with partially conflicting results. It is still unclear which specific parts of the neurological examination can be reliably performed in virtual settings, and despite some general guidance and recommendations for the remote neurological examination [18,22], there is no relative updated review focusing on movement disorders. 

Given the accumulating literature evidence, in this review, we aim to summarize and discuss recent evidence on the feasibility and reliability of the different components of neurological examination in remote settings via videoconferencing and suggested adaptations, focusing on the examination of patients with movement disorders, which may be useful for both general and subspecialty neurologists. In addition, based on the available evidence and research gaps, we provide additional insights that could stimulate future research.

Although our purpose was not to conduct a systematic review, we followed a systematic search methodology; we searched the MEDLINE database for peer-reviewed articles written in English, investigating the feasibility and reliability of neurological examination in patients for movement disorders via videoconferencing, with no time restrictions. We used the following terms in different combinations: “telemedicine”, “telecare”, “telehealth”, “remote”, “virtual”, “video”, “videoconference”, “videoconferencing”, “movement disorders”, “Parkinson’s disease”, “parkinsonism”, “hyperkinetic”, “hypokinetic”, “Huntington’s disease”, “dystonia”, “tremor”, “tic”, “tics”, “chorea”, “atypical parkinsonian”, “multiple system atrophy”, “corticobasal”, “progressive supranuclear palsy”, “dyskinesia”, “neurological examination”, “neurological exam”, “bradykinesia”, and “rigidity”. The bibliography of each relevant article was also screened in order to detect possible additional studies and include the key recent evidence. Our search was conducted between June 2023 and August 2023. Our conclusions were based on the available literature evidence in combination with our 2-year experience with the “Outpatient Clinic for Memory, Dementia and Parkinson’s disease through the National Telemedicine Network” for patients in the Aegean islands in Greece [23]. 

## 2. General Aspects for the Telemedicine Visit

**Figure 1:**
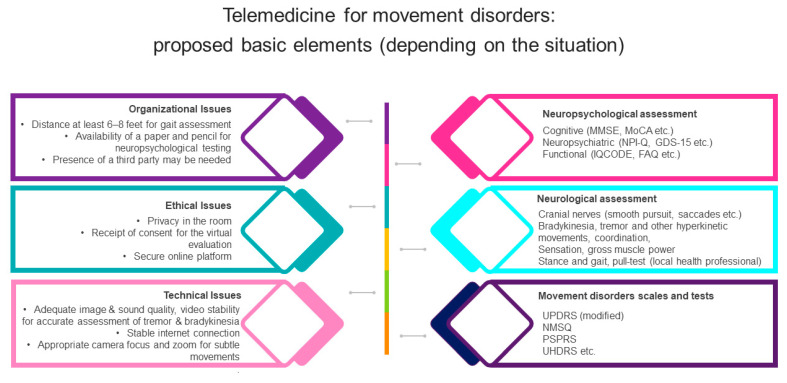
Proposed basic elements (depending on the situation) of a telemedicine visit for movement disorders.

The American Academy of Neurology (AAN) has provided a general guidance and recommendations about the setup of teleneurology visits, as well as the performance of the basic parts of neurological examination [22,24,25]. At the beginning of the telemedicine visit, the patient’s name and date of birth should be verified, and consent for the virtual examination should be received [18] (Figure 1). The room should be private, well lit, close to the internet router, and large enough for the observation of gait [22]. The patient should be comfortably sitting in a chair, and paper and pencil are suggested to be available for the examination [22]. Video stability, adequate image and sound quality, no noise interference, and appropriate camera focus should be also ensured. The exact location and address of the patient should be recorded, in case of any emergent situation [26]. 

Concerning the appropriate videoconferencing software, a recent review has provided some commercially available options for movement disorders, considering the technical and data security characteristics, as well as compliance with US and European regulations [27]. The online platform and network should be ensured to be secure, and for privacy reasons, the healthcare professionals should not be located in open-plan offices unless they wear headphones during the videoconferencing [6]. 

The opportunity of multidisciplinary care is an important advantage of telemedicine, as various healthcare specialists can be invited and participate during the videoconferencing, thereby contributing to optimal management. For the assessment of patients with movement disorders, an ideal care team could include a primary care physician, a neurologist, a geriatric psychiatrist, a neuropsychologist, a nurse, a physical therapist, a speech pathologist, and a social worker, depending on the situation [22,28]. 

Since muscle tone, reflexes, strength, sensation, and balance evaluation cannot be reliably evaluated remotely, according to the report of the telemedicine work group of the AAN, the presence of the local physician is necessary for a complete neurological examination via telemedicine [24]. Alternatively, a trained registered nurse, physician assistant, or other healthcare professional could perform these parts of the neurological examination under the supervision of the remote neurologist specialist in front of the camera [24,29]. If the examination is performed at home, a family member or caregiver could facilitate the physician–patient communication, give insight into the patient’s answers, and assist with specific parts of the remote examination, such as holding or moving the camera [18,28]. Furthermore, the body language of the caregiver/family member might also guide the clinician for specific questions and give deeper clinical insights [28]. If possible, after the obtainment of consent from the patient, the physician could arrange a phone call with the caregiver/family member before and after the telemedicine visit in order to gather useful information [28]. The presence of a third party is even more important in case of cognitive impairment or psychiatric manifestations (Table 1) [28]. 

## 3. Neurological History through Telemedicine

Neurological history is considered the most significant part of the neurological assessment, and its importance is further highlighted in remote settings, including the case of movement disorders. Although there is no study specifically investigating the reliability of video-based neurological history, a survey among teleneurology providers indicated that the remote obtainment of neurological history was perceived as the same or better compared to the in-person encounter, for various subspecialties including movement disorders [30]. 

However, several issues related to the virtual environment might impact the process of physician–patient communication and neurological interview, which could subsequently influence the quality of neurological assessment. In particular, patient-related factors, such as hearing impairment, have been identified as barriers during the virtual interaction with PD patients via video, which could result in limitations in understanding and effective interaction during the neurological interview [31]. Technical aspects, such as low audio quality, disrupted internet connection, and audio delays for some seconds during the real-time videoconferencing might impact the interactive communication between the patient and the physician [32,33]. Simultaneous speaking has been also shown as a barrier during virtual communications between PD patients and clinicians in another study [34], which could also affect the process of obtaining the neurological history. However, a study investigating speech and voice parameters in PD patients showed that participants and clinicians could successfully compensate for these kinds of disruptions by adequately waiting for the other party to finish talking before answering [32]. Prior familiarization of the patients and family members and/or caregivers with the use of the equipment, the provision of clear, written instructions to the participants before the initiation of the telemedicine visit, and a back-up plan in case of technological breakdown have been proposed as potential solutions, to ensure successful connection, use, and access to the online platform, which could facilitate the communication [28]. The lack of intimacy, the poor quality of interactive engagement, the lower confidence towards the clinician, and frustration with technical issues that have been mentioned by some PD patients as limitations in virtual visits in another study [31] may also intervene with the neurological interview. PD patients have also stated that they felt more limited in expressing themselves and asking questions compared to in-person medical visits [31]. Physicians have also reported that technical issues such as having to restart the computer, connection difficulties, the need to reset accounts, and screen freezing during telemedicine visits with PD patients were very distracting and bothersome [31]. On the other hand, higher levels of convenience and comfort, feelings of being understood, and greater satisfaction compared to in-person encounters have been also reported [31]. It is still unclear if and how these factors could impact the neurological history of patients with movement disorders, and future studies are needed towards this direction.

## 4. Neurological Examination via Videoconferencing

### 4.1. Cognitive, Neuropsychiatric, and Functional Assessment

The examination of cognitive function is an integral part of the neurological assessment of patients with movement disorders including PD, HD, atypical parkinsonian syndromes, and others, depending on the clinical scenario. In cases in which there is a trained healthcare professional or neuropsychologist at the originating site, the appropriate neuropsychological and neuropsychiatric batteries can be administered a priori and then sent to the telemedicine team [28] (Table 2).

The number of studies investigating the reliability of remote neuropsychological testing in patients with movement disorders is limited. The Montreal Cognitive Assessment (MoCA) is a widely used screening method for patients with PD [35] and evaluates eight cognitive domains: attention and concentration, orientation, memory, language, calculations, executive function, conceptual thinking, and visuoconstructional skills, and its administration lasts about 10 min [36]. It has been demonstrated that the remote administration of MoCA in patients with PD and HD with mild cognitive deficits via web-based videoconferencing is feasible, while barriers included disrupted images and delays in sound due to poor internet connection [37]. In some cases, due to inadequate lighting and the presence of tremor, clear screenshot images could not be easily obtained, as patients could not steadily place the paper in front of the camera to show the cube drawing sample, for example [37]. In agreement with this evidence, another study indicated that MoCA in PD patients is feasible via videoconferencing at their homes, with the use of available technological equipment and online platforms (e.g., Skype) [38]. In the first study, the items of MoCA that required physical drawing were sent to patients via e-mail, while they were asked to print them immediately before the telemedicine examination. In the second study, a sealed envelope with a printed copy of the first three items of MoCA was given to the patients. In the first case, only patients with an available printer could participate in the study, while in the second case, MoCA scores might have been artificially better through the videoconferencing, because of assistance or previous rehearsal. Web-based MoCA versions and touchscreens might eliminate these limitations. A blind version of MoCA can also be used in cases of severe vision impairment or low video quality [22]. Concerning other screening neuropsychological tests, it has been demonstrated that the remote administration of the Mini Mental State Exam (MMSE) is valid in the elderly population, as well as in individuals with dementia [39], suggesting that it could possibly be reliable in the case of movement disorders as well, although further evidence is required to validate this hypothesis. 

For the neuropsychological examination of distinct parts of cognition, studies in PD are lacking. However, in other populations, several studies have confirmed the reliability of the remote performance of a wide range of neuropsychological tests, suggesting their potential validity for PD as well. In this context, as reviewed recently, existing literature evidence supports the relative reliability of remote performance of the Digit Span task (Digit Span Forwards, Digit Span Backwards, and Digit Span Total) for attention and working memory, the Oral Trails A test for processing speed, the full 60-item and the shorter 15-item Boston Naming Test (BNT), letter and category fluency for language, the Hopkins Verbal Learning Test-Revised (HVLT-R) including the HVLT-R Delayed Recall for memory, and the Clock Drawing Test for visuospatial and executive skills [40]. For the assessment of executive function, the Frontal Assessment Battery (FAB) and the similarities subtest (WAIS-IV) can be also performed remotely, while for the evaluation of visual cognitive function, the Cube Copying Test and Short-form JLO are also feasible through videoconferencing [41]. 

During the remote performance of neuropsychological testing, it should be ensured that patients do not write down the items for recalling or ask for help from other individuals in the room. For this purpose, they should be asked to angle the camera at the desk and to not take notes during memory testing. In addition, microphones should be on high volume, so the examiners can hear if a third party assists the patient during the assessment [42]. 

For the neuropsychiatric assessment during videoconferencing, subtle signs of a potential psychiatric co-pathology can be observed, such as darting eyes, paranoia surrounding the remote neurological examination, close relatives or the caregiver, withdrawal, and disorganized speech [28]. For the assessment of depressive symptomatology, it has been demonstrated that the Geriatric Depression Scale (GDS-15), the Hamilton Depression Rating Scale (HDRS), and the Patient Health Questionnaire-9 (PHQ-9) can be administered reliably remotely [41,43]. Informants can also provide information for remote completion of the Neuropsychiatric Inventory Questionnaire (NPI-Q), as well as the Mild Behavioral Impairment (MBI) Checklist (MBI-C) [41]. 

Regarding the functionality, high intra- and inter-rater reliability have been indicated between in-person and remote assessment of the activities of daily living (ADL) via the motor part of the Functional Independence Measure (FIM) in patients with PD [44]. The lowest agreement was observed for the FIM item of bowel management, which was attributed to the different interpretation and scoring of patients’ responses [44]. Informant-based functional instruments that could also be used remotely include the Informant Questionnaire on Cognitive Decline in the Elderly (IQCODE), the Functional Activities Questionnaire (FAQ), and the 4-item instrumental ADL scale [41].

### 4.2. Examination of Cranial Nerves

If smell deficits, like anosmia or hyposmia, are mentioned during the neurological history, or a synucleinopathy, mainly PD or DLB, is suspected, a detailed testing of cranial nerve I function, such as the University of Pennsylvania Smell Identification Test (UPSIT), can be formally performed remotely if available, with the assistance of a local healthcare professional. Alternatively, the UPSIT test can be posted to patients, to ask them to send their answers electronically, a process that has already been performed in the PREDICT-PD study [45]. 

Cranial nerve II is rarely examined during the assessment of patients with movement disorder complaints. Visual acuity (VA) cannot be reliably assessed remotely, and fundoscopy cannot be conducted during videoconferencing. However, if there is a concern about visual impairment, VA symmetry could be imprecisely examined by asking the patient to read the same words by covering each eye separately, while holding a newspaper or a book at a steady distance from the eyes [18]. Visual fields cannot be reliably evaluated via videoconferencing, but a suggested screening technique is the following: the patient is asked to move at a two-foot distance from the monitor of a computer, and look directly with each eye separately at a pen or another object that the physician holds in front of the camera [18]. If the four corners of the monitor can be seen by the patient, then a large field cut, such as hemianopia, can be rather excluded, whereas smaller deficits at the periphery of the fields can be missed [18]. 

For the assessment of cranial nerves III, IV, and VI, the patient should be asked to move his/her face close to, and fixate on, the camera, and any pupillary asymmetry, nystagmus, ptosis, or ocular misalignment can be identified in the primary position [18]. The frequency of macro square wave jerks, which would be useful for the assessment of suspected progressive supranuclear palsy (PSP), for example, might be especially difficult to detect through video. For ocular movement assessment, smooth pursuit can be performed remotely by asking the patient to follow his/her own or the caregiver’s/family member’s finger in front of the camera, while manually elevating the upper lids during downward gazing [18,22]. For the detection of nystagmus, the patient can be requested to stay for a few seconds at each position of gaze [18]. For the examination of horizontal and vertical saccadic eye movements, the patient should alternately look between the top left and right corners, and above and below the screen respectively [18], while manually elevating his/her upper lids for the observation of downwards saccades. Alternatively, the patient can look between the center of the camera and an item at home in each of the four cardinal directions [22]. The slow velocity of vertical saccades, as observed in PSP, may be particularly challenging to assess through video. Appropriate lighting, speed of internet connection, and adequate quality of the video are required for the observation of eye movements [22]. For the examination of vestibulo-ocular reflex (VOR)—as needed for the assessment of supranuclear palsy in the case of suspected PSP for example—the patient should be asked to focus on the camera or the face of the physician and move the head up and down, as well as side to side [22]. 

For the examination of cranial nerve V and in cases of facial sensory complaints, the physician can ask the patient to use a tissue or a cold object, like an ice pack, and check for asymmetry in sensation at each of the trigeminal nerve distributions [18]. Atrophy in temporalis muscles and jaw deviation during mouth opening can also be noted [18]. Facial asymmetry and nasolabial fold flattening can be detected via video. The patient can be asked to squeeze eyelids with eyes tightly closed, raise eyebrows, blow out cheeks, purse lips, smile, and show teeth in front of the camera [18]. Hypomimia and reduced spontaneous eyeblinking can be effectively detected during the video-based telemedicine visit. It has been noted that technical issues, like background lighting, low contrast, shadowing on the face, pixelation of the image, and absence of zoom function of the camera, could influence the assessment of facial symmetry and movements [32], suggesting that enhanced focus and zoom camera functions might alleviate these limitations. Body and head dystonia, as well as camptocormia, have also been mentioned as additional factors restricting the view of the patient’s facial features [32]. As formal hearing assessment cannot be performed via video [18], if hearing impairment is mentioned by the patient, an in-person examination by an otorhinolaryngologist should be arranged. For a gross assessment, the patient or the caregiver/family member can be instructed to perform bilateral finger rub and compare the two sides [22]. 

For cranial nerves IX, X, and XII, during the neurological history and videoconferencing, hypophonia, nasal speech, spastic dysarthria, scanning, or any other speech abnormality can be identified [18]. The physician can also ask the patient to phonate “ta”, “ka” and “pa”. Regarding hypophonia, it has been shown that low audio quality may cause the clinicians to ask patients to repeat themselves in order to be well heard, despite the use of lapel microphones [46]. However, according to the researchers of this study, although the time was increased with this process, it did not seem to affect the accuracy of the assessments. Another study has indicated that the majority of video-captured speech and voice parameters of PD patients assessed asynchronously by trained speech–language pathologists was reliable compared to the in-person evaluation [32]. These parameters included speech intelligibility in conversation and reading, overall articulatory precision, pitch range, vowel prolongation duration, vocal tremor, perceptual breathiness ratings, lack of clarity, phonation breaks, lip and tongue movement, and sound pressure levels. If there is enough lighting and video quality, the symmetry of the elevation of the soft palate can also be noted [18]. For the evaluation of swallowing and pharyngeal function, the patient can be requested to drink a small sip of water and also to demonstrate a strong cough [22]. If the resolution of the video is adequate, the patient should be asked to stay close to the camera, and then tongue atrophy and fasciculations can also be observed [18]. Finally, the physician can ask the patient to protrude his/her tongue and move it rapidly from side to side [18]. 

Depending on the clinical scenario, for cranial nerve XI, the physician can ask the patient to expose his/her neck and shoulders and then search for trapezius and sternocleidomastoid muscle atrophy [18]. For a gross evaluation of muscle strength, the patient can be asked to shrug his/her shoulders and look to the right and left [18]. 

### 4.3. Motor Examination

Dependent upon the clinical case and quality of the video, relevant body parts can be exposed and observed for atrophy and fasciculations [18].

One important limitation of the remote neurological examination is the inability to examine muscle power precisely, including the use of the Medical Research Council (MRC) scale, unless a trained healthcare professional is present during the videoconferencing [18]. However, at least antigravity power can be grossly observed by asking the patient to perform arm abduction, flexion and extension at the elbows, wrist and finger flexion and extension, index finger abduction, as well as thumb abduction and extension [18]. For the lower extremities, the camera might be required to move downwards, and the patient in a sitting position should straighten and raise one knee at a time off the chair, as well as dorsiflex and plantarflex the feet [18]. Functional strength maneuvers can be used for the evaluation of muscle groups [22]. The patient can be asked to stand up from a sitting position with arms folded in front of the chest, while caution is needed for the camera to adequately capture the movement. Dependent on the clinical concern, heel and toe raises, as well as jumping, can be requested for the evaluation of dorsiflexion and plantarflexion muscle power [22]. 

Tone, deep tendon reflexes, and superficial reflexes including plantar responses cannot be reliably examined virtually in the absence of a trained physician or registered nurse [18]. Family members might be instructed to perform the patellar reflex and plantar response with a spoon or spatula in cases of relative familiarity with this process [22]. Pyramidal signs might be identified by the examination of pronator drift, finger tapping (see below), or tapping the thumb alternatively with each finger [18]. The inability to examine rigidity remotely represents a major limitation for the assessment of patients with suspected parkinsonism. In these cases, the presence of a physician or another trained healthcare professional at the originating site is of paramount importance [47]. A study investigating the feasibility of the use of telemedicine for at-home titration in cases of LCIG infusion has demonstrated that most neurologists felt no significant limitations during the virtual evaluation of the patients, compared to hospital in-person assessment, as they could visually estimate postural instability and rigidity during the mobility of the patient [48]. However, in the free text of the relative questionnaires, clinicians mentioned the inability to reliably determine rigidity and perform “pull-test” in this study [48]. These results suggest that although rigidity cannot be examined remotely, it might not have significant effects on the overall neurological assessment of PD patients, at least for the case of titration in LCIG. 

For the detection of tremor and other hyperkinetic movement disorders, the patient should be asked to stay still with his/her arms at rest on his/her legs and then raised in front of him towards the camera. The physician should observe for any abnormal postures suggesting dystonia, or any involuntary movements, like tremor, dystonia, chorea, myoclonus, or tics [18]. In order to evaluate the upper and lower limbs, as well as the torso, caution is needed for the camera to be repositioned if required in order to capture the entire body. In a qualitative study among PD patients and physicians who participated in video-based telemedicine, visits showed that in some cases the feet and outstretched arms of the patients could not be viewed, because the camera was too close to them [31]. For eliciting stimulus-sensitive myoclonus, a caregiver/family member could be possibly instructed to perform the task.

Tremor is the most frequent hyperkinetic movement disorder, and the accurate discrimination between tremor phenotypes (resting, postural, kinetic, intention, and orthostatic) is crucial for establishing a syndromic approach, diagnosis, and treatment. Resting tremor can be evaluated through video while the patient is seated in front of the camera with his/her hands relaxed on his/her thighs [18]. It can also be detected during the video-based neurological interview, although the patient might retain a position that suppresses its manifestation, such as holding their hands in their lap. Resting tremor can be revealed by distraction, such as counting backwards from one hundred, by subtracting one each time or mentioning the months of the year backwards, which can be also performed remotely [18]. The “pill-rolling” character of parkinsonian tremor can be detected through video, although video quality and resolution might limit its assessment. Postural tremor can be observed by outstretching the hands upfront or by abducting the arms at the shoulders and flexing them at elbows while the hands are pronated at the “wing-beating” position in front of the chest for a few seconds. The examination of kinetic tremor can be performed by finger-to-nose testing (see below), asking the patient to draw the Archimedes spiral on a piece of paper and then showing it to the physician via the camera, or by pouring water between two cups [18]. A printed version of the Archimedes spiral can also be sent via e-mail or downloaded online, so the patient can draw a line by following that of the given drawing. It is recommended for patients to angle the camera, in order for the neurologist to ensure that the spiral is being drawn without placing their arms on the desk [42]. The hard copies of the spiral drawings can be sent via e-mail to the telemedicine team. A digital method of analysis of the Archimedes spiral has also been used, in which spiral width, length, and the total distance performed by the pen during the drawing are computed [42]. For the assessment of kinetic tremor—and micrographia as well—the patient can also be asked to provide a writing sample in front of the camera, and a screenshot can be made [18]. A tablet and a digital stylus can also be used for sharing a digital writing or spiral sample, in cases in which the patient is familiarized with technology [22]. Due to the rhythmic element of tremor, video resolution per frame and the quality of network connection may affect its evaluation, so the optimal video quality should be ensured especially in these cases [11]. Alien limb phenomena, as in cases of corticobasal syndrome (CBS), can also be observed through video.

Bradykinesia can be reliably evaluated remotely, provided that video quality is adequate and the network connection is stable. For the examination of upper limbs, the patient should sit in a chair in front of the camera, and for lower limbs, caution is needed for the camera to be able to capture the lower half of the body. For finger tapping, one hand at a time, the patient should be asked to bend the elbow, raise the hand with the arm unsupported, and tap the index finger and thumb as quickly as possible, with the largest possible amplitude between the two fingers [18]. Next, the physician can ask the patient to open and close each fist, as well as pronate and supinate each forearm fully, rapidly and repetitively [18]. For foot and toe tapping, the camera may be required to be placed looking downwards, although desktop computers might not allow for this adjustment. The patient should be requested to tap the foot and toes on the floor quickly and repetitively maintaining a large distance from the floor [18]. In case of adequate room space, bradykinesia can also be examined during the gait assessment via video, by observing the decreased arm swing and its symmetry and the reduced length of stride [18]. As mentioned above, during the remote neurological interview, hypomimia and reduced spontaneous eyeblinking can be detected, while micrographia can also be identified by asking the patient to show a writing sample in front of the camera.

It has been considered that the use of appropriate lighting or camera angles is crucial for the remote assessment of dystonia, especially in cases when its features are very focal or task-specific [11]. Caution is also needed for the placement and zoom of the camera, since it should be able to capture even subtle postures or movements of lower limbs.

### 4.4. Sensory Examination

The inability to perform a formal sensory examination is another limitation of the remote neurological assessment. As a screening evaluation through video, the patient can be asked to use a household object (like a tissue) and a cold item (like a spoon under cool running water or ice pack) in order to check for symmetric sensation between index fingers and big toes [18]. Sensory ataxia can be tested by asking the patient to raise the arms, close the eyes, and touch the nose separately with each finger [18]. Although the reliability of cortical sensory examination (stereognosis, graphesthesia, 2-point discrimination) via telemedicine is unknown, the caregiver/family member could be possibly instructed to perform the task, especially in cases of relative complaints or a suspected CBS.

### 4.5. Coordination

Detection of scanning speech and nystagmus testing have been described above. For the assessment of dysmetria—but also kinetic tremor—the patient should be asked to fully extend their arms and then touch the nose with the index finger (finger-to-nose testing) close enough to the camera [18]. As another option, the patient can hold an item, like a pen, in front of them, and then alternatively reach the object and nose with the index finger of the contralateral side [18]. Alternatively, the patient can be requested to move the finger between the nose and the camera or a fixed item at home [22]. If a family member, caregiver, or assistant is present during the telemedicine examination, they can hold their index finger in front of the patient in different locations, in order to perform the finger-to-nose exam [18]. For the heel-to-shin examination, the patient has been proposed to place each leg on an ottoman or stool in front of him and then perform the maneuver as usual. For dysdiadochokinesia testing, rapid alternating movements can be performed as for in-person visits [18]. Rebound phenomenon cannot be examined via video, unless a trained healthcare professional is present. During gait assessment, wide-based or staggering gait can also be observed (see below). 

### 4.6. Stance and Gait

Depending on the room size and the camera setup and angle, gait assessment might be unable to be properly assessed [18]. It has been suggested that the distance between the patient and the camera should be at least 6–8 feet away for the entire body visualization, which, however, might intervene with the assessment of subtle tremor or dystonia, hypomimia [26]. Hence, the patient might be required to move during the examination, according to the requirements of each examination part, as well as reposition the camera facing downwards to a hallway [22]. 

First, stance with feet together can be observed at rest. Then, the patient can be asked to walk preferably at least five steps from one side of the room to the other in both directions, and also from close to the camera to away from it [18]. Similarly, tandem gait can also be evaluated, but the presence of a family member or another third party close to the patient while performing this activity would be recommended for safety reasons. Precautions should be taken in order to diminish fall risk, and if applicable, patients should be asked to use their assistive device [26]. Among 550 telemedicine visits of patients with PD performed during a two-year period, one fall was documented [26]. As it has been mentioned in a qualitative study among PD patients and physicians who were involved in video-based neurological assessments, if the patient cannot walk unassisted and there is no third party in the room, the gait will not be able to be assessed [31]. Space limitations may also inhibit the assessment of freezing of gait [26]. The physician should pay attention to any subtle limb tremor or dystonia during gait assessment, since these deficits might be easily missed via video-based examination, especially if the video quality is poor. It has been mentioned that several awkward readjustments and maneuvers may be required in some cases during the neurological examination of some patients, in order for the lower limb and posture to be assessed [31]. Due to the risk of falling and subsequent injury, the Romberg test and “pull test” are not recommended to be performed remotely unless a trained healthcare professional is close to the patient [1,18]. In selected cases, and after safety is ensured, the patient can be instructed to perform the Romberg test while the third party is close [22].

## 5. Movement Disorders’ Scales via Telemedicine

### 5.1. Parkinson’s Disease

The most well-established and widely used tool for the neurological assessment of disease severity and progression in PD is the Unified Parkinson’s Disease Rating Scale (UPDRS), as well as the Movement Disorder Society (MDS) UPDRS (MDS-UPDRS). In particular, part III of UPDRS and MDS-UPDRS is considered the gold standard evaluation method for the motor signs of patients with PD [49] (Table 3).

#### 5.1.1. UPDRS

Although most items of the UPDRS can be virtually performed, the examination of rigidity and postural instability via retropulsion testing necessitate in-person evaluation. A modified version of incomplete UPDRS lacking the parts of rigidity and retropulsion pull testing (items 22 and 30) has been validated for remote administration via video, both cross-sectionally and longitudinally at 2 years [50]. The results were similar for patients with PD under levodopa and pramipexole treatment, suggesting that the type of dopaminergic therapy might not influence the observed validity [50]. However, in this study, any potential variability between in-person and remote assessments because of video-related factors, including differences between 2D and 3D perception or positioning that might affect the reliability of the remote UPDRS, has not been evaluated [50]. Furthermore, only patients with early PD were included [50], limiting the generalizability of the results for patients with advanced PD or levodopa-induced motor complications. Another recent study has developed an equation which is able to predict with high accuracy the global severity of motor symptoms based on partial UPDRS part III scores, lacking rigidity and postural instability items that cannot be assessed online [51]. As this study was based on in-person assessments, future studies should investigate the agreement between the predicted UPDRS part III score obtained from partial scores via video-based examination and total UPDRS III score assessed via in-person visits.

Evidence on direct comparisons between in-person and remote UPDRS assessment dates back to 1993, when Hubble and colleagues had already reported that the motor subscale of UPDRS could be evaluated via videoconferencing, with no significant individual differences in rating between the in-person and remote physicians who independently examined the patients at the same day [52]. In this study, a nurse clinician familiarized with PD assisted in rigidity assessment [52]. Low internet speed, poor video quality, and increased cost of the technological equipment were crucial limitations during that early period of telemedicine use. In a randomized controlled trial, UPDRS rating was also reliable via videoconferencing compared to one-time simultaneous in-person assessment of patients with PD [44]. Another randomized controlled trial aiming to assess the feasibility of specialized care provided by movement disorder specialists through videoconferencing for patients with PD has demonstrated that the motor subscale of UPDRS with the assistance of a trained nurse for the conduction of pull testing and the assessment of rigidity is valid and reliable compared to the in-person evaluation [53]. In this study, the performed pull testing by the local nurses was observed via video and rated remotely by the movement disorder specialists, while rigidity testing was verbally relayed to the specialists for assessment [53]. Rigidity and leg agility were the only items not displaying fair agreement between the telemedicine and in-person evaluations [53]. Notably, a subgroup analysis excluding patients with levodopa-induced motor fluctuations demonstrated that the agreement for rigidity and leg agility assessment was improved [53]. In agreement with this evidence, UPDRS part III was also reliable through a web-based platform compared to in-person evaluations [54]. Hence, even though this cannot be considered as an ideal substitute for the neurologist’s in-person examination [52], if a trained nurse or other healthcare professional is present during the telemedicine examination, rigidity and postural instability assessment for the UPDRS motor subscale may be also relatively reliably performed even for patients with motor fluctuations. 

Concerning the reliability of the virtual administration of different parts of UPDRS part III, a study has indicated lower agreement between in-person and video-based assessment of tremor and bradykinesia by the same raters, compared to gait and postural instability [55]. Since the evaluation of bradykinesia involves tasks with continuous and repetitive movements, it has been hypothesized that technical issues such as low video quality due to disrupted network connection, time lags and blur, image pixelation or freezing in motion might significantly influence its rating. For instance, because of a blurry image, a rater should ask the patient to slow down while performing the finger tapping testing in one study, which could have an influence on the neurological assessment [6]. Furthermore, the multiple components of bradykinesia scoring in the UPDRS could potentially create additional complexity compared to the evaluation of balance and gait [1]. Global bradykinesia might also be difficult to assess since it necessitates the visualization of the entire body of the patient, so it can be underestimated [26]. The rhythmic character of tremor, as well as the measurement of its amplitude in centimeters, might also affect its assessment via video [1]. However, there is also evidence that the examination of tremor and motor speed in patients with PD is valid [56], and subtle signs can still be evaluated through video [45]. Speed and quality of internet connection have been proposed to be important factors for the assessment of bradykinesia and tremor in particular, so potential technical differences might explain the differences in these results [1,6]. A study investigating the influence of filming conditions via digital camera video clips on the assessment of movement disorders, including tremor, demonstrated that quality of images had a critical effect on tremor assessment, while the use of a tripod did not [57]. The kind of device may also play a role, since the video quality and resolution may be generally worse via a tablet or smartphone compared to a desktop computer, making quick and subtle involuntary movements like tremor of small amplitude or mild dyskinesia difficult to observe [58]. On the other hand, desktop computers with built-in cameras might not allow for the visualization of the entire body including the lower limbs [6]. Technological advances may limit these barriers in the future. Furthermore, another study demonstrated that among the different parts of UPDRS regarding the quality of life, handwriting exhibited relatively low agreement between the remote and in-person assessment [44]. The low image resolution due to internet connection bandwidth issues in the rural areas could result in pixilated images, which might have led to unreliable interpretation of the handwriting samples [44]. Of concern, the parts of lower limb examination, including toe tapping, leg agility, and rest tremor, were the most commonly missed items during telemedicine visits of patients with PD [26], further highlighting the importance of the camera placement for complete neurological assessment.

In addition to the UPDRS part III, substantial agreement has been demonstrated between the web-based and in-person performance of the patient-administered scales UPDRS part II, which evaluates the motor experiences of daily living, and IV, which assesses motor complications [54]. 

#### 5.1.2. MDS-UPDRS

Although the validity and reliability of the remote MDS-UPDRS compared to the in-person assessment has not been investigated, its feasibility has been confirmed in several cases [26]. A virtual version of MDS-UPDRS without the rigidity and retropulsion items has already been used in a study with remotely enrolled patients with PD, for the confirmation of diagnosis [59]. It has been demonstrated that up to three missing values can be tolerated in order to obtain a valid total surrogate score for the motor subscale of MDS-UPDRS across all Hoehn and Yahr stages [60]. Based on this evidence, Goetz and colleagues highlighted that a virtual version of MDS-UPDRS part III cannot be reliably used, since the rigidity and retropulsion testing items are more than three, and further noted the need for future studies to validate the remote version of MDS-UPDRS compared to in-person evaluation [61]. Notably, significantly lower total MDS-UPDRS part III scores have been demonstrated among video raters compared to in-person raters in another study, and subgroup analyses showed that bradykinesia and tremor sum of scores were those of the greatest disagreement [55]. This evidence suggests that more subtle deficits evaluated with the MDS-UPDRS motor subscale might not be easily detected via video assessments.

A recent study using exhaustive computational searching methodology has identified a shortened 8-item version of the MDS-UPDRS with high agreement with the original MDS-UPDRS that can be administered remotely [47]. In particular, the researchers of this study initially excluded the items of rigidity and retropulsion testing, as well as those that require the inspection of lower limbs (toe tapping, leg agility, gait, freezing of gait, posture, and body bradykinesia), since during remote examination patients usually use a digital device (smartphone, tablet, or computer) placed in front of them on a table or desk [47]. This proposed abbreviated MDS-UPDRS version for the remote assessment included the following items: 1.13 fatigue, 2.5 dressing, 2.10 tremor, 2.12 walking and balance, 3.2 facial movements, 3.4 finger tapping, 3.9 rising from chair, and 4.3 time spent in the off state [47]. The shortened MDS-UPDRS version requires only 5–10 min to complete, which is much shorter than the 30 min of the original MDS-UPDRS. This advantage is even more significant for the telemedicine settings, given the additional barriers, such as the potential need for repositioning of the camera and the difficulties in observing the various body parts, especially the lower extremities [47]. In this study, the total MDS-UPDRS was evaluated, while future studies could focus on the motor subscale, or the daily living aspects, since these parts might be more relevant depending on the clinical purposes. 

Collectively, although the validity of the remote motor subscale of MDS-UPDRS needs further investigation, it seems that a trained healthcare professional would be required for the assessment of rigidity and postural instability, in order to obtain an adequate and reliable virtual rating. In addition, caution is required for subtle signs of bradykinesia and tremor, since the assessment of these elements is more challenging during the telemedicine visits.

#### 5.1.3. Other Scales for Parkinson’s Disease

It has been demonstrated that the video-based physical assessment of patients with PD through several tests was feasible with high accuracy levels via a tele-rehabilitation system, compared to in-person clinical evaluation [46]. In particular, the timed stance test, step test, the Timed “Up and Go” test (TUG), steps in 360° turn, lateral and functional reach, as well as Berg Balance Scale could be remotely performed with relatively high reliability [46]. For the performance of the TUG, pre-visit instructions can be sent to patients, in order to measure a distance of 10 ft in the room, and then place a chair and a tape marker at the two ends [42]. During the telemedicine visit, the caregiver or family member can hold the camera, so the examiner can view the walking area [42]. 

Furthermore, the self-filled PD quality of life scale-39 (PDQ-39) and Non-Motor Symptom questionnaire (NMSQ), as well as the physician-administered Rush Dyskinesia Rating scale (RDRS), have shown substantial agreement between the web-based and in-person administration [54], suggesting that the assessment of the severity of levodopa-induced dyskinesias, non-motor symptoms, and quality of life can also be reliably performed remotely. The RBD Screening Questionnaire (RBDSQ) has also been already used remotely in the PREDICT-PD study [45]. 

### 5.2. Atypical Parkinsonian Syndromes

The number of studies on movement disorders other than PD is limited. For PSP, a study has recently created two modified versions of the PSP Rating Scale (PSPRS), a well-established measure for assessing the clinical severity of the disease, which can be administered remotely [62]. The first 25-item version lacks the items of limb and neck rigidity and postural instability, while the second 21-item version additionally lacks the ocular motor and limb dystonia items, given the assumption that the assessment of these parts might be less reliable via video and more dependent on internet connection or video quality [62]. Both scales showed excellent agreement with the original PSPRS, at baseline, and at 6 and 12 months, suggesting that these versions could also predict disease progression [62]. Importantly, the presence of a caregiver is essential during the assessment of this scale, in order to adjust the position of the camera, bring water for swallowing evaluation, and ensure the safety of the patient for the gait examination [62]. Another study has demonstrated good inter-rater reliability for the motor part of a videotaped version of PSPRS—without rigidity—that was scored by several independent raters [63]. In this study, the less reliable items were dysphagia and limb dystonia. 

Good diagnostic agreement has been recently revealed between self-reported and video-based diagnoses of atypical parkinsonian syndromes (multiple system atrophy (MSA), PSP, CBS, and dementia with Lewy bodies) of patients enrolled in The Michael J. Fox Foundation’s Fox Trial Finder [64]. In this study, a remote investigator assessed the patient during a real-time recorded telemedicine visit, which was subsequently reviewed by a second investigator. Both raters were blinded to the self-reported diagnosis of the participants. The highest diagnostic concordance was observed for the self-reported diagnosis of PSP, while the lowest for CBS [64]. The lower diagnostic agreement for CBS could be possibly explained by the fact that CBS diagnosis relies on a rather more detailed neurological examination, including the evaluation of asymmetric rigidity, cortical sensory and motor symptoms (apraxia, cortical sensory loss and alien limb syndrome), pyramidal signs, and myoclonus, whose remote assessment could be particularly challenging or impossible. Although the examination of postural instability cannot be remotely evaluated and the examination of ocular motor impairment is difficult, the neurological history of early falls and the characteristic “look of surprise” could provide adequate cues for video-based PSP diagnosis, which could explain the highest diagnostic concordance in this case. However, since an in-person neurological assessment was not performed in this study, further evidence is needed on the reliability of the video-based examination of patients with atypical parkinsonian syndromes. 

### 5.3. Essential Tremor

A recent study has demonstrated substantial agreement for the assessment of tremor diagnosis and severity between the remote and standard in-person video recordings of the neurological examination of patients with essential tremor and healthy controls [65]. The video-based diagnosis in both in-person and remote conditions was also in agreement with the intake diagnosis for most participants [65]. Importantly, the agreement was lower for milder cases of tremor, suggesting that smaller tremor amplitudes might be less observable and quantifiable in remote settings [65]. Hence, the lower fidelity of the video-based assessment for cases with milder tremor could be more detrimental, potentially resulting in misdiagnosis [65]. Concerning the technical aspects, the resolution of the camera for both the in-person and remote video recordings, as well as the speed of internet connection, were not correlated with higher agreement levels [65]. Although tremor rating was mentioned to be difficult due to the low video quality of some remote recordings, the overall high diagnostic agreement indicates that the physicians could effectively compensate for these technical challenges [65]. There were also some missing video parts, because of the inadequate familiarization with technology in some cases, which supports the important role of training before the telemedicine examination [65]. Regarding the remote administration of commonly used scales for ET, the validation of the Essential Tremor Rating Assessment Scale (TETRAS) and Fahn–Tolosa–Marin Clinical Rating Scale for Tremor (FTM) has not been formally performed for remote use [11]. 

### 5.4. Huntington’s Disease

Similar to the modified version of the motor subscale of the UPDRS for patients with PD, a modified video-based form of the motor component of the Unified Huntington’s Disease Rating Scale (UHDRS) without the balance and rigidity testing has been shown to be reliable compared to the in-person evaluation for patients with Huntington’s disease (HD) [15]. Test–retest reliability of the virtual assessment of motor scores was demonstrated to be excellent. Interestingly, the highest agreement between the remote and in-person evaluation was for bradykinesia and total chorea, while oculomotor and dystonia assessment displayed the relatively lowest levels of agreement in this study [15]. The visualization of the whole body is generally required for the examination of patients with HD in order to observe potential subtle features, such as chorea in the toes [15]. These signs may be harder to detect via telemedicine, where the upper body part is usually visible [15]. In addition, the assessment of saccades necessitates a high-resolution camera, which might not always be available [15]. Even with these limitations, the remote neurological examination of patients with HD is a valuable tool, especially for the follow-up in previously diagnosed individuals.

### 5.5. Cervical Dystonia and Other Dystonia Syndromes

Cervical dystonia is characterized by involuntary sustained or intermittent muscle contractions of the neck muscles causing abnormal, often repetitive, movements and/or postures. Botulinum toxin injections in the affected muscles is the treatment of choice, which requires ongoing neurological reassessment of its efficacy, while patients’ self-reports during therapeutic intervals are possibly unreliable [17]. The Toronto Western Spasmodic Torticollis Rating Scale (TWSTRS) is a validated measure for evaluating the severity of cervical dystonia, while it represents an ideal candidate for remote use since it does not necessitate hands-on examination [66]. A recent study demonstrated excellent agreement for the motor severity subscale of the Toronto Western Spasmodic Torticollis Rating Scale (TWSTRS) between remote and in-person evaluations of patients with cervical dystonia [17]. Among the specific items of the TWSTRS motor subscale, range of motion and retrocollis severity did not reach the threshold for at least a moderate level of agreement. During the telemedicine visit, the patient is asked to alter his/her position, while during the in-person medical visit, the physician can freely rotate the patient, potentially resulting in a more reliable evaluation [66]. This study suggests that the video-based TWSTRS could be reliably performed remotely; however, given the fact that the same clinician conducted the in-person and remote TWSTRS ratings, future studies with multiple raters are needed.

Oromandibular dystonia is a focal dystonia, presenting as involuntary masticatory and/or lingual muscle contractions. It is frequently misdiagnosed as a functional movement disorder, bruxism, or temporomandibular disorder, resulting in inappropriate management. Interestingly, a multilingual website for oromandibular dystonia, via which patients can complete questionnaires, send images or videos capturing their involuntary movements, and participate in videoconferencing via Skype, has been shown to facilitate the remote diagnosis of this disorder [67]. 

### 5.6. Tourette’s Syndrome and Other Tic Disorders

Behavioral treatments for Tourette’s syndrome and persistent (chronic) motor or vocal tic disorder (PTD) have been shown to be beneficial for the reduction of tic severity [68]. However, these treatments are often unavailable, especially in rural areas where there is a lack of trained specialists. A recent pilot randomized controlled trial demonstrated that parent-guided and therapist-guided internet-delivered behavior therapies for young patients with Tourette’s syndrome or PTD aged 8–16 years were feasible and acceptable by patients and parents [69]. In this study, the Yale Global Tic Severity Scale (YGTSS)—a clinician-administered scale measuring the severity of motor and vocal tic symptoms and tic-associated impairment—was administered at baseline and follow-up visits, some of which were conducted via videoconferencing or telephone, depending on personal preferences, travel distance, or technical reasons [69]. In a randomized controlled clinical trial evaluating the effectiveness of therapist-supported online behavioral intervention for tics in young patients, the YGTSS was administered remotely during the follow-up visits [70]. Therefore, the YGTSS can be performed via both video and telephone, facilitating the remote assessment of tic severity of children and adolescents. 

### 5.7. Tardive Dyskinesia 

Tardive dyskinesia (TD) is a movement disorder associated with the long-term use of dopamine-receptor blocking agents for the treatment of psychiatric conditions such as schizophrenia, major depression, and bipolar disorder [71]. It is characterized by abnormal, repetitive, and involuntary movements of the face and body, which may be persistent even after ceasing the relevant medication [71]. A study among patients with at least 10-year exposure to antipsychotics has demonstrated that the conduction of the Abnormal Involuntary Movement Scale (AIMS), a commonly used instrument for assessing TD, via videoconferencing was reliable compared to the in-person evaluation [72]. A recent survey among neurology and psychiatry physicians who remotely assessed patients with drug-induced movement disorders including TD showed that 28% and 19% of them found it challenging to observe gait and tics/movement difficulties through video, respectively [71]. Video-based assessment may not enable the visualization of the entire body, while patients often attempt to hide or control their movements when they are observed [73]. These challenges might affect the reliability of the overall virtual evaluation.

## 6. Discussion and Future Directions

In summary, a growing body of evidence supports the feasibility of most parts of the remote neurological examination of patients with movement disorders. In particular, the assessment of stance and gait—in case an assistive device is not required—bradykinesia, tremor, dystonia, some parts of ocular mobility, coordination, and a gross assessment of muscle power and sensation is possibly feasible via video, even if a third party is not present during the telemedicine examination. Technical issues, including the video quality, internet connection, and the placement of the camera, might affect the detection of bradykinesia, tremor, or dystonia, especially in mild cases. Due to the inherent character of rigidity, postural instability, and tendon reflexes testing that requires hands-on examination, these maneuvers cannot be reliably performed unless a trained healthcare professional is present. In selected cases, the patient or a third party can be instructed to assist in the performance of some elements of the exam, including specific parts of cranial nerves, sensation, and Romberg testing. Finally, although studies in patients with movement disorders are scant, most parts of the neuropsychological testing can be remotely performed with high reliability, including screening instruments like MoCA.

The standardization of the remotely administrated scales and tests is critical for maintaining their reliability and validity in telemedicine settings. Some validation studies have confirmed that specific scales (UPDRS, TUG, etc.) can be administered remotely with comparable results to in-person assessments with minor adaptations. However, given the large diversity in telemedicine settings regarding the technological equipment, connectivity issues, healthcare professionals’ experience, and patients’ familiarity with technology, standardized protocols for the remote neurological and neuropsychological assessment in movement disorders are needed [74]. Given that the quality of internet connection, image, and sound may affect the accuracy of the remote evaluation, specific minimum requirements, such as the use of high-definition cameras and stable internet connectivity, should be ensured. The development of standard operating procedures (SOPs), including workflows and clinic templates, the steps of obtaining informed consent, troubleshooting of technical failures, and using secure platforms for the telemedicine session, is also recommended. As mentioned above, private, large enough and well-lit rooms for minimizing potential distractions and enabling the full view of the patient are also important. Furthermore, a step-by-step guide with detailed instructions and the essential modifications for the remote administration, scoring, and interpretation of each scale should be developed, and appropriate training for healthcare professionals who are going to administer these tests through telemedicine is also needed for enhancing inter-rater reliability and ensuring consistency [75]. Standardization measures should also consider the different movement disorders, the disease stage, as well as cognitive function, since these factors may significantly affect the accuracy of remote assessments. Finally, standardized and validated tools for evaluating patients’, caregivers’, and healthcare professionals’ satisfaction with the telemedicine services are also needed. These standardization strategies will enable the comparability of results from relative studies, thereby supporting the growth of telemedicine. However, although these measures can provide the fundamental principles for the diagnostic and curative medical procedures, they should be adapted to each patient and family, according to the situation and their specific biological, psychological, cultural, and social needs [76]. 

In most countries, there are no official requirements regarding the experience and training of healthcare professionals for practicing telemedicine. Healthcare providers with less years of experience, as well as those dealing with multiple sclerosis, movement disorders, cognitive disorders, and headache, were more likely to mention that an in-person assessment would not have affected the medical plan, compared to a telemedicine evaluation [30]. Given the additional challenges of telemedicine, it can be proposed that an experienced, senior movement disorder specialist might be more suitable for remote visits compared to a less experienced one, although additional factors, such as flexibility, adaptability, empathetic and caring personality traits, and communication skills would also be important. 

A global survey among members of the Movement Disorder Society (MDS) demonstrated that about 75% of respondents would be interested in telemedicine education [20]. Some movement disorder academic centers have developed and use online training programs for healthcare professionals who will be performing remote assessments, and upon completion, they receive certification to practice via telemedicine [77]. A telehealth skills curriculum has also been developed based on the Association of American Medical Colleges (AAMC) for the conduction of telehealth objective structured clinical evaluations (OSCEs), which includes several competency domains: interpersonal and communication skills such as maintaining eye contact, showing concern and care, and paying attention to verbal and non-verbal cues, professionalism such as assisting patients in using the technological equipment and confirming confidentiality, skills in patient assessment and care such as history taking and providing clear instructions during the remote physical examination, as well as medical decision-making, such as reviewing red flags for urgent symptoms, clarifying follow-up arrangement, and confirming mutual understanding for diagnostic and therapeutic planning [75]. Telesimulation has also been proposed as a promising method for providing practical training across a range of the various telehealth competencies [78], and it can also be applied in the field of movement disorders. 

Importantly, not all patients with movement disorders would be suitable for remote evaluation, while health-related, technological, and practical factors should be considered when selecting patients for telemedicine assessments. Good candidates would be those requiring regular follow-up for assessing response to symptomatic treatment and side effects, especially related to dopaminergic therapy, those with levodopa-induced dyskinesias and motor fluctuations necessitating a close monitoring, as well as those with a known diagnosis and relatively stable condition without unexpected clinical deterioration. From a technological perspective, patients with reliable and stable internet connection, comfort using technology and devices, as well as those with supporting family members or caregivers willing to assist with technical issues would also be ideal candidates. In addition, patients living in remote and underserved areas lacking movement disorder specialists, as well as those with severe mobility and transportation difficulties, are expected to benefit the most from telemedicine consultations.

Concerning the neurological examination, given the fact that rigidity is a key clinical feature of the diagnosis of parkinsonism, and that UPDRS is not recommended for diagnostic purposes, UPDRS can be used remotely mainly for assessing the severity of symptoms in patients who have been previously examined in person and have received a diagnosis of PD, in case a trained healthcare professional is not present at the originating site [47]. The inability to assess rigidity remotely becomes an even more important limitation among patients in whom this is a major clinical manifestation [1]. 

Notably, given the inability to examine sensation, tendon reflexes, and muscle power in detail remotely without the assistance of a trained healthcare professional, potential other causes of patients’ symptoms such as imbalance would be possibly missed. For instance, the diagnosis of peripheral neuropathy or cervical myelopathy that require a detailed sensory and motor examination cannot be reliably made via video. In the same vein, potential other causes of progressive disability of already diagnosed patients with PD or other movement disorders might be also missed [1]. In these cases, the neurologists who remotely assess the patient need to have high clinical vigilance for asking an in-person assessment. Furthermore, if there are concerns about a potentially serious medical condition, the physician should ensure that an urgent in-person neurological assessment can be performed [18]. In-person evaluation may be needed in cases of unexpected clinical deterioration, the emergence of atypical characteristics, or uncertainty regarding differential diagnosis. 

In particular, patients with severe or rapidly progressing movement disorders may necessitate an in-person visit, allowing for a more thorough assessment. Initial diagnostic assessments for patients with movement disorders are also more likely to require an in-person visit. Atypical clinical manifestations may require in-person evaluation for a detailed neurological and neuropsychological examination, structural and functional neuroimaging studies, as well as laboratory or genetic testing, to ensure diagnostic accuracy. For instance, patients with suspected CBS may require in-person evaluation for an accurate diagnosis, since some components of the neurological examination, such as cortical sensory deficits and pyramidal signs, are more difficult to assess via telemedicine. Patients with suspected PD, displaying bradykinesia and not evident resting tremor, may also require in-person evaluation for diagnosis, in order to assess muscle tone. 

Studies examining the diagnostic agreement for the differential diagnosis of movement disorders, such as PD versus ET or atypical parkinsonian syndromes, as well as ET versus dystonia, are also scant. In addition, it would be important for future studies to investigate the contribution of neuroimaging or laboratory findings (e.g., DaTSCAN, brain magnetic resonance imaging (MRI)) to the remote neurological assessment of patients with movement disorders, as well as if and to what extent these findings may affect agreement between the in-person and remote diagnoses.

Importantly, it has been demonstrated that the environment in which PD patients are examined—at their home or in a clinic—might affect the severity of parkinsonian symptoms [54], suggesting that the settings of videoconferencing could additionally influence the clinical evaluation of these patients. It can be hypothesized that the telemedicine examination might be viewed by patients as frightening or distracting, resulting in increased stress and anxiety, thus affecting patients’ motor status, including the possible exacerbation of tremor [52]. However, patients might perceive any medical visit as intimidating or become stressed because of the burdensome travel to the neurologist’s office for an in-person visit in an urban area [52]. These external factors are difficult to completely eliminate or precisely evaluate, not only during the remote telemedicine visit but also the in-person examination [52]. 

Existing evidence suggests that the neurological assessment of patients with PD through video is feasible either at patients’ homes or doctors’ offices [1]. Tremor, bradykinesia, rigidity, and dyskinesia might limit the ability of the patients to move the camera or use the devices [6]. In the case of home-based video assessments, space limitations might restrict the visualization of the entire body of the patient, while a family member could possibly aid in setting up the equipment or repositioning the camera [1]. This is particularly important for the assessment of gait, lower limb function, the global movement spontaneity, and the constancy of resting tremor [6]. However, only desktop computers without moveable cameras might be available in some cases, which creates further limitations [6]. This difficulty could be overcome by asking the patients a priori to be placed in a room large enough for enabling walking and the observation of the entire body [6]. Another crucial factor is the fact that a trained physician or other healthcare professional in healthcare facilities would significantly facilitate the neurological examination and the possible performance of some parts like rigidity or postural instability assessment. However, an advantage of the video assessment at a patient’s home is the opportunity to observe the environment and potentially propose alterations for safety, such as preventing the risk of falls. 

Available evidence about the efficacy of telemedicine in movement disorders derives from studies with a relatively short-term follow-up; longitudinal studies are needed in order to investigate the long-term reliability and effects of the remote neurological evaluation. In addition, in cases of non-randomized clinical trials, patient self-selection is another important limitation, since it would more likely be for individuals more familiar and comfortable with the use of technology to participate, leading to selection bias [6]. In addition, in most studies, raters were healthcare professionals who had received training in performing the movement disorders’ scales used and technical advice. Therefore, the generalizability of these results to real-world situations warrants caution. It has been proposed that even the most experienced movement disorder specialists would rather need training in the remote MDS-UPDRS [26]. Hence, real-world evidence on the reliability of the remote neurological examination of patients with movement disorders is necessary in order to obtain meaningful information for routine clinical practice.

It has been considered that the assessment of potential functional neurological disorders might be more challenging via telemedicine [79]. Relatively fair inter-rater agreement has been identified among movement disorder specialists for the diagnosis of psychogenic jerks [80], as well as on the judgment between an organic or functional movement disorder based on video interpretation [81]. The differences in accuracy between phenomenologic and etiologic diagnostic assessments reflect the importance of the neurological history and complete neurological examination for the comprehensive clinical assessment [82]. On the other hand, telemedicine gives the opportunity for a more detailed observation of the patient at their homes or other environments, which could assist in the differential diagnosis of functional movement disorders. Nevertheless, further research is needed to identify the reliability of diagnosis of functional movement disorders synchronously through video. 

Emerging evidence suggests that the objective measurement of rigidity or postural instability through wearable sensors might at least partially compensate for the inability to examine these elements remotely [1]. For instance, rigidity scores of UPDRS could be predicted by wearable sensors in approximately 85% of patients with PD, and postural instability can be effectively identified by wireless accelerometers [83]. A recent study has also demonstrated that the automated measurement of postural instability and lower limb impairment of PD patients via an optical RGB-Depth device is feasible and in agreement with the UPDRS examination by neurologists [84]. Therefore, a combination of video-based neurological assessment and the use of digital sensors may represent a promising method of the remote evaluation of PD patients [1]. Novel digital sensors can also aid in the objective quantitative evaluation of tremor [85] and levodopa-induced dyskinesias [11], thereby paving the way for future research combining the use of videoconferencing and data from wearable sensors. 

In addition, artificial intelligence could possibly aid in the neurological assessment of patients with movement disorders, which could further support the findings of the remote examination. In this context, a recent study using an artificial intelligence algorithm has demonstrated that a conventional webcam-based technology could detect and quantify bradykinesia in the upper limbs of PD patients with high diagnostic accuracy, based on speed, amplitude, and fatigue [86]. Interestingly, visual data extracted from other patients’ movements have been correlated with rigidity, such as the reduction in arm and leg swing during the assessment of gait [87], and specific characteristics of stance have been associated with postural instability [84]. Based on this concept, a recent study combining the red, green, and blue (RGB) computer vision algorithm and machine learning with available visual motor features from the MDS-UPDRS part III developed a remote scoring predictive model for estimating rigidity and postural instability in PD with high accuracy [88]. Therefore, these clinical features that cannot be assessed virtually might be able to be estimated via machine learning models, whose accuracy could be further improved in the future.

An important limitation of most of the abovementioned studies is the fact that they have included patients who are relatively young, familiarized with technology, and at early stages of PD [1]. However, age, the stage of PD, cognitive impairment, educational level, and other comorbidities like hearing or visual difficulties might possibly affect the validity of neurological assessment through video. In this context, it has been indicated that the diagnosis of PD based on videotaped UPDRS assessment was more likely to be incorrect in cases of lower scores in bradykinesia, resting tremor, and action tremor, as well as shorter duration of symptoms, suggesting that milder parkinsonian symptoms and earlier stages of PD might be more difficult to diagnose via video [89]. On the other hand, the video-based diagnosis did not significantly differ from in-person evaluations in terms of age, levodopa dose, Hoehn and Yahr score, posture, gait, or hypomimia scores of the patients [89]. In addition, it has been mentioned that telemedicine visits can be effectively performed for PD patients of older age and significant disability, and in these cases the remote assessment would probably be more valuable, given the mobility difficulties and challenges related to travel experienced by these subgroups [26]. Future studies should consider these additional factors and include a broader population of PD patients.

The remote neurological assessment of patients with movement disorders will also enable the wider participation of patients of dispersed geographical areas in clinical trials, as well as the longitudinal follow-up for longer time periods [8]. In addition, patients living in rural underserved areas and suffering from genetic forms of PD, such as GBA or LRRK2 mutations, can be monitored remotely without the need for traveling long distances to see a PD specialist [8]. 

Several ethical issues should be considered in telemedicine, to ensure patients’ welfare and safety. The fundamental principles of nonmaleficence and beneficence, as well as respect for patients’ rights and dignity, should be applied in telemedicine settings as well [90]. Patient privacy, confidentiality, and data security are of paramount importance, and the platforms should be Health Insurance Portability and Accountability Act (HIPAA)-compliant [90]. In addition, the patient and the remote physician should be in private rooms. Given the limitations and challenges of the remote assessment, informed consent is another important ethical consideration, and it should be ideally obtained in a written form from the patient or a legal representative. The patient should be informed about the nature, benefits, and risks of the telemedicine visit, and the possibility that an in-person assessment might be required for reaching an accurate diagnosis. Although telemedicine aims at mitigating healthcare disparities, digital illiteracy and the lack of the appropriate technological equipment and connectivity may result in additional inequities, particularly for older individuals and those with financial difficulties [91]. Furthermore, standardized practice guidelines should be developed, in order to ensure high-quality remote healthcare delivery [90]. The professional–patient relationship is another important ethical aspect that should be considered in telemedicine settings; the remote physician should demonstrate empathy, professionalism, and a caring attitude.

The use of telemedicine may result in significant cost savings for patients, caregivers, and healthcare systems. Telemedicine visits for patients with PD have been associated with lower travel distances and time [92], which may lead to reduced direct travel expenses, but also indirect savings, such as days out of work and caregiving time. Moreover, the mobility challenges that often accompany patients with movement disorders may be associated with additional costs. Telemedicine also facilitates the more efficient distribution of healthcare resources and enables timely medical evaluation, possibly leading to reduced hospital admissions and visits to the emergency departments, which may result in cost-effectiveness and increased savings for the healthcare system. Through telemedicine, patients with movement disorders in remote areas have better access to specialized care, possibly leading to more effective management and long-term savings for the patient and the healthcare system. 

The integration of telemedicine assessments with in-person care, when possible, could offer a “best of both worlds” approach, benefiting from the stated advantages of telemedicine while compensating for the shortcomings that have been mentioned, namely the limited neurological examination. A hybrid model combining both means of care could enhance the accuracy of the diagnosis, provide the opportunity for thorough investigation with neuroimaging or laboratory tests, certain treatments, as well as strengthen the connection between patient and physician by their in-person interaction. According to Furuya and colleagues, the hybrid model constitutes a service consisting of alternating in- person and telemedicine assessments, with new patient visits being virtual, and the initial follow-up visits scheduling in person [93]. This proposed model is based on the assumption that new appointments are dedicated to a great extent to obtaining a detailed medical history, which can be relatively easily performed remotely. During the initial follow-up in-person visit, a problem-focused neurological exam can be conducted, as well as a diagnostic work-up. Subsequent assessments can be conducted in person or via telemedicine, depending on the situation and needs of the patient, whenever required. One important advantage of the hybrid model is that it enables screening to avoid unnecessary transportation, as well as potentially preventable hospital admissions. The remote neurological assessment could occur at a patient’s home, with the simultaneous presence of the patient’s primary health physician, who could assist during the conduction of the neurological examination and communication with the patient [94]. This would demand a higher administrative load, requiring both physicians’ and the patient’s availability, but it would completely remove the need for the patient’s relocation to the local hospital for example. For maintaining continuity of care, patient’s data collected through a telemedicine visit should be securely stored in electronic health records, which should be accessible to the other healthcare professionals involved in the patient’s care.

Difficulties stemming from technical issues, such as unstable internet connectivity and low image and sound quality, are well-recognized barriers to the widespread adoption of telemedicine. New technological developments, however, have significantly mitigated these challenges. Video cameras with high framerates and high video resolution can provide an accurate depiction of the patient, while also capturing fine movements such as tremor. This is further aided by the ability of newer cameras to magnify and focus on the area of interest, using optical zoom. Internet connectivity can be improved with the adoption of 5G technology, which offers higher bandwidth and lower latency [95]. Additionally, newer video compressing formats (Versatile Video Coding, VVC or H.266) have outperformed older standards in terms of compression efficiency and promise to deliver higher video quality with lower data usage [96]. As this trend of technological developments continues, the gap between in-person visits and telemedicine is expected to keep diminishing.

Collectively, based on the existing literature evidence, ideal patients with movement disorders eligible for video-based neurological assessment could be those living in remote underserved areas or with ambulation difficulties, with a diagnosis established previously in person, with a caregiver or family member available for participating in the telemedicine visit, with high-speed internet connection and technological equipment of adequate quality, as well as willingness to be assessed remotely. If the telemedicine visit is carried out in a medical center, a trained healthcare professional could significantly assist in the assessment of rigidity, postural instability, and possible other parts of the examination unable to be performed virtually. In addition, interventional therapies requiring frequent reassessments constitute good candidates for follow-up via telemedicine. Caution is needed for subtle clinical features that are difficult to evaluate virtually, such as mild tremor or dystonia, new emerging symptoms, and concerns in differential diagnosis, since in-person examination may be needed for accurate assessment. Nevertheless, in cases of restricted access to medical care due to the lack of specialists or long distances, even the partial virtual neurological examination would be arguably preferred compared to no examination at all.

## 7. Conclusions

In conclusion, telemedicine can be a valuable supportive tool for the specialized neurological assessment of patients with movement disorders for specific indications, especially those living in remote underserved areas or suffering from mobility difficulties. Although the video-based neurological examination cannot completely replace the hands-on neurological assessment and clinical vigilance is needed in order to determine the cases requiring in-person evaluation, technological advancements and ongoing research will significantly aid in its appropriate and safe use in the clinical settings.

## Figures and Tables

**Table 1:** Proposed basic organizational and technical elements of a telemedicine visit for patients with movement disorders.

Basic Aspects of a Telemedicine Visit for Patients with Movement Disorders	Proposed Elements
Organizational issues	Verification of patient’s name, date of birth, and address
	Availability of a paper and pencil close to the patient
	Ideally a multi-disciplinary team (primary care physician, neurologist, psychiatrist, neuropsychologist, nurse, physical therapist, social worker, depending on the situation)
	Presence of a trained healthcare professional is needed for a complete neurological examination
	A third party (family member or caregiver) could facilitate the communication
	Distance between the patient and the camera should be at least 6–8 feet away for the entire body visualization for gait assessment
Ethical and Privacy Issues	Privacy in the room
	Receipt of consent for the virtual neurological evaluation
	Secure online platform
Technical issues	Well-lit room and close to the internet router
	Video stability, adequate image and sound quality
	Stable internet connection
	No noise interference
	Appropriate camera focus and zoom function
	Avoidance of background lighting, low contrast, and shadowing on the face
	Caution is needed for the placement of the camera, especially for the examination of lower limbs

**Table 2:** Proposed elements of the neurological assessment of patients with movement disorders through telemedicine.

Neurological Assessment of Patients with Movement Disorders through Telemedicine	Proposed Elements (Depending on the Situation)
Cognitive assessment	Montreal Cognitive Assessment (MoCA)
	Mini Mental State Exam (MMSE)
	Boston Naming Test (BNT)
	Letter and category fluency
	Hopkins Verbal Learning Test-Revised (HVLT-R)
	Clock Drawing Test (CDT)
	Frontal Assessment Battery (FAB)
	Similarities subtest (WAIS-IV)
	Cube Copying Test
	Short-form JLO
Psychiatric assessment	Geriatric Depression Scale (GDS-15)
	Hamilton Depression Rating Scale (HDRS)
	Patient Health Questionnaire-9 (PHQ-9)
	Neuropsychiatric Inventory Questionnaire (NPI-Q)
	Mild Behavioral Impairment (MBI) Checklist (MBI-C)
Functional assessment	Informant Questionnaire on Cognitive Decline in the Elderly (IQCODE)
	Functional Activities Questionnaire (FAQ)
	4-item instrumental ADL scale
	Functional Independence Measure (FIM)
Examination of cranial nerves	Remote performance of University of Pennsylvania Smell Identification Test (UPSIT) if available and a local physician is present or if being posted
	Gross assessment of visual acuity by reading words in a newspaper covering each eye separately
	Gross assessment of visual fields by focusing on an item held by the physician and looking at the corners of the screen
	Assessment of smooth pursuit by following one’s own finger
	Assessment of saccades by asking the patient to look alternatively at the top left, right, above, and below the screen
	Vestibulo-ocular reflex can be assessed by focusing on the camera and moving the head up and down and side to side
	Assessment of facial sensation asymmetry with a tissue or cold object Hypomimia, reduced eye blinking, facial asymmetry can be noticed
	Asking to squeeze eyelids with eyes tightly closed, raise eyebrows, blow out cheeks, purse lips, smile and show teeth in front of the camera
	Gross assessment of hearing by performing bilaterally finger rub and comparing the two sides
	Hypophonia, nasal speech, spastic dysarthria, scanning can be noticed
	Strong cough demonstration, drinking a small sip of water
	Atrophy of trapezius and sternocleidomastoid muscle, shrugging shoulders and looking to the right and left
	Tongue protrusion and moving from side to side
Motor examination	Gross assessment of at least antigravity power by arm abduction, flexion, and extension at the elbows, wrist and finger flexion and extension, index finger abduction, thumb abduction and extension
	Functional strength maneuvers for muscle groups
	Tone, deep tendon reflexes, and superficial reflexes can be assessed if a trained healthcare professional is present
	Pyramidal signs examined by pronator drift and finger tapping
	Resting tremor can be assessed while sitting with the hands relaxed on the thighs and also by distraction
	Postural tremor can be assessed by outstretching the hands upfront or by abducting the arms at the shoulders and flexing them at the elbows
	Kinetic tremor can be assessed by finger-to-nose testing, drawing of the Archimedes spiral on a piece of paper and showing it to the physician via the camera, or by pouring water between two cups
	Dystonia, myoclonus, tics, chorea, alien limb phenomena can be noticed
	Bradykinesia can be assessed as usual
Sensory examination	Symmetry sensation assessment with a cold item or a tissue
	Sensory ataxia can be tested by asking the patient to raise the arms, close the eyes, and touch the nose separately with each finger
	For the cortical sensory examination (stereognosis, graphesthesia, 2-point discrimination), a third party could be possibly instructed to perform the task
Coordination	Finger-to-nose testing
	The patient can be requested to move the finger between the nose and the camera or a fixed item in the room
	Rapid-alternating movement as usual
Stance and gait	Stance with feet together can be observed at rest
	Asking the patient to walk preferably at least five steps from one side of the room to the other in both directions
	For tandem walking, a third party is recommended to be present for safety reasons
	Pull-testing can be performed if a trained physician is present

**Table 3:** Movement disorders’ scales and tests that can be used via telemedicine.

Movement Disorder	Scales and Tests
Parkinson’s disease	Modified versions and equations of Unified Parkinson’s Disease Rating Scale (UPDRS) without rigidity and postural instability
	Timed “Up and Go” test (TUG)
	Steps in 360° turn
	Berg Balance Scale
	PD quality of life scale-39 (PDQ-39)
	Non-Motor Symptom questionnaire (NMSQ)
	Rush Dyskinesia Rating scale (RDRS)
	RBD Screening Questionnaire (RBDSQ)
Atypical parkinsonian syndromes	Modified versions of Progressive supranuclear palsy (PSP) Rating Scale (PSPRS)
Huntington’s disease	Modified version of the motor component of the Unified Huntington’s Disease Rating Scale (UHDRS) without the balance and rigidity items
Cervical dystonia	Toronto Western Spasmodic Torticollis Rating Scale (TWSTRS)
Tourette’s syndrome and other tic disorders	Yale Global Tic Severity Scale (YGTSS)
Tardive dyskinesia	Abnormal Involuntary Movement Scale (AIMS)
